# Mucus

**Published:** 1868-08

**Authors:** Andrew S. Cutler


### ARTICLE IV.

### *Mucus.*

By Andrew S. Cutler. D. D. S.

This is a secretion peculiar to internal lining membrane. The term itself is rather an indefinite one, and is applied in general to the viscid, effused matter found upon all mucous surfaces, as, for instance, the mouth, nose, lungs, alimentary canal, &c. When the body is in a state of health, the secretion of true mucus is exceedingly small in amount, and difficult to obtain for experimental purposes. In many forms of disease, however, it is poured out more freely. But, an abnormal condition of the system. must imply a vitiated state of the secretion also. Hence, one difficulty in obtaining it pure. The chief difference between diseased and healthy mucus is undoubtedly owing, principally, to the greater proportion of albuminous and epithelial matter found in the former. Mucus, ordinarily, is a clear colorless fluid of remarkable viscidity. Its peculiar characteristic is determined by a principle termed mucosin. This substance is insoluble in water, but may be readily precepitated by the action of different acids. Various animal fluids also have the faculty of diluting it to a certain extent. Mucus seems made up, chiefly, of epithelial scales, with certain globular substances of a rather indefinite nature, floating in a perfectly transparent menstruum. It seems notedly susceptible to decomposition, yielding the usual products of animal decay.

Mucus seems to be homologous in general characteristics with the skin, hair, and nails, which, indeed, are undoubtedly but peculiar modifications of it. The particular, function of this secretion, seems to be, for the most part, physical. Acting, as it does, as a protection to the surfaces over which it is effused, and in combination with other fluids, facilitating the passage of foreign substances through cavities, where unless some similiar provision were made, an inflamed action would unavoidably result. As before intimated, the difficulty of obtaining normal, healthy mucus, in a pure state, for analytical purposes, must be great, and may be owing, First, To the exceedingly small amount secreted in a state of health. Secondly, To the great trouble experienced in separating it from the epithelial cells thrown out from the tissues. And thirdly, To the presence of contained albuminous matter, existing in a state of peculiar chemical combination with the very principle to be got at. There are also a variety of other intricacies requiring careful consideration. So that the final results, when obtained, are only approximatively correct. Mucocin, or Mucin, the nitrogenous principle of mucus may be rendered soluble by dilute alkalies. Its ultimate analysis into simple elements, discovers a large proportion of carbon, with an ample percentage of nitrogen to imply rapid putrefactive changes. The following table is from Scherer.

| | |
|---|---|
| Carbon | 52.41 |
| Hydrogen | 6.97 |
| Nitrogen | 12.82 |
| Oxygen | 27.80 |
| | 100.00 |

The subjoined is an analysis by Berzelius of healthy nasal mucus.

| | |
|---|---|
| Water | 930.7 |
| Mucin | 53.3 |

| | |
|---|---|
| Alcohol extract, and alkaline lactates......... | 3.0 |
| Chlorides of sodium and potassium............ | 5.6 |
| Water extract, with traces of albumen and phosphates.......................................... | 3.5 |
| Soda, combined with mucus.................... | 3.9 |
| | 1000.0 |

Jacubowitsch found an analysis of buccal mucus like the following.

| | | | |
|---|---|---|---|
| Water........................................................ | | | 990.02 |
| Solid Matter. | Organic matter soluble in alcohol | 1.67 | |
| | " " insoluble " " | 2.18 | |
| | Fixed Salts.......................... | 6.13 | 9.98 |
| | | | 1000.00 |

